# Effect of sub-chronic exposure to cigarette smoke, electronic cigarette and waterpipe on human lung epithelial barrier function

**DOI:** 10.1186/s12890-020-01255-y

**Published:** 2020-08-12

**Authors:** Baishakhi Ghosh, Hermes Reyes-Caballero, Sevcan Gül Akgün-Ölmez, Kristine Nishida, Lakshmana Chandrala, Lena Smirnova, Shyam Biswal, Venkataramana K. Sidhaye

**Affiliations:** 1 Department of Environmental Health and Engineering, Johns Hopkins Bloomberg School of Public Health, Baltimore, MD USA; 2 Department of Environmental Health and Engineering, Center for Alternatives to Animal Testing, Johns Hopkins Bloomberg School of Public Health, Baltimore, MD USA; 3 Present Address: Department of Pharmaceutical Toxicology, Faculty of Pharmacy, Marmara University, Istanbul, Turkey; 4 Division of Pulmonary and Critical Care Medicine, Johns Hopkins School of Medicine, Baltimore, MD USA; 5 Department of Mechanical Engineering, Johns Hopkins Whiting School of Engineering, Baltimore, MD USA

**Keywords:** E-cigarette, Cigarette, Nicotine, E-cadherin, Waterpipe, Tobacco, Cilia

## Abstract

**Background:**

Taking into consideration a recent surge of a lung injury condition associated with electronic cigarette use, we devised an in vitro model of sub-chronic exposure of human bronchial epithelial cells (HBECs) in air-liquid interface, to determine deterioration of epithelial cell barrier from sub-chronic exposure to cigarette smoke (CS), e-cigarette aerosol (EC), and tobacco waterpipe exposures (TW).

**Methods:**

Products analyzed include commercially available e-liquid, with 0% or 1.2% concentration of nicotine, tobacco blend (shisha), and reference-grade cigarette (3R4F). In one set of experiments, HBECs were exposed to EC (0 and 1.2%), CS or control air for 10 days using 1 cigarette/day. In the second set of experiments, exposure of pseudostratified primary epithelial tissue to TW or control air exposure was performed 1-h/day, every other day, until 3 exposures were performed. After 16–18 h of last exposure, we investigated barrier function/structural integrity of the epithelial monolayer with fluorescein isothiocyanate–dextran flux assay (FITC-Dextran), measurements of trans-electrical epithelial resistance (TEER), assessment of the percentage of moving cilia, cilia beat frequency (CBF), cell motion, and quantification of E-cadherin gene expression by reverse-transcription quantitative polymerase chain reaction (RT-qPCR).

**Results:**

When compared to air control, CS increased fluorescence (FITC-Dextran assay) by 5.6 times, whereby CS and EC (1.2%) reduced TEER to 49 and 60% respectively. CS and EC (1.2%) exposure reduced CBF to 62 and 59%, and cilia moving to 47 and 52%, respectively, when compared to control air. CS and EC (1.2%) increased cell velocity compared to air control by 2.5 and 2.6 times, respectively. The expression of E-cadherin reduced to 39% of control air levels by CS exposure shows an insight into a plausible molecular mechanism. Altogether, EC (0%) and TW exposures resulted in more moderate decreases in epithelial integrity, while EC (1.2%) substantially decreased airway epithelial barrier function comparable with CS exposure.

**Conclusions:**

The results support a toxic effect of sub-chronic exposure to EC (1.2%) as evident by disruption of the bronchial epithelial cell barrier integrity, whereas further research is needed to address the molecular mechanism of this observation as well as TW and EC (0%) toxicity in chronic exposures.

## Background

Personal devices that deliver aerosolized nicotine for recreational purpose, known as Electronic Nicotine Delivery Systems or electronic cigarettes (EC) use an electric heating element to vaporize a liquid mixture (e-liquid) to produce an aerosol. Therefore, many potential harmful constituents from the combustion of tobacco in cigarette smoke (CS) are absent in EC. Thus, EC are currently accepted as a healthier alternative to ordinary cigarettes [1]. While long-term effects of EC in people are not known, animal studies have demonstrated that there are toxic effects of EC [2, 3], and in vitro studies have found some dysregulation in bronchial and lung epithelial cells, lung fibroblast, and alveolar macrophages [2].

Tobacco smoking is the primary risk factor of chronic obstructive pulmonary disease (COPD) development. COPD is a progressive illness caused by chronic lung injury, with clinical manifestations that can include bronchitis, airway reactivity, and emphysema [4]. Considering the increased incidence of e-cigarette or vaping product use-associated lung injury, the evaluation of the biological activity of EC aerosol in in vitro models has become a top priority [5]. Hence, we studied the effects of sub-chronic EC aerosol exposure on human bronchial epithelial function in an in vitro air-liquid interface model in comparison to CS, and tobacco waterpipe (TW) exposures, in similar conditions. As the lining of the respiratory tract, the epithelia form a physical barrier that effectively prevents inhaled environmental insults from damaging the sub-epithelial layers [6]. Not surprisingly, we and other researchers have demonstrated that CS exposure disrupts the connectivity between adjacent cells in the lung epithelium interfering with the function of cell-cell adhesion proteins, often members of the cadherin family of adhesion molecules [7–12]. Additionally, there is evidence of detrimental effects of CS exposure on the mucociliary clearance of entrapped particulates in the lung track, which is mediated by the synchronous beats of protruding organelles in ciliated cells from the lung epithelium [13, 14]. Thus, our study informs on the effects of EC aerosol exposure on the lung epithelial function and a synergistic effect of nicotine in the e-liquid. We demonstrated that EC aerosol, as well as CS and TW can distinctly harm the lung epithelial barrier, an observation that in the context of previous experiments, supports a correlation with the time of exposure. Therefore, our report emphasizes the need for more studies of long-term health and harmful consequences that may affect users of ECs.

## Methods

### Study design

We investigated the epithelial cell barrier function deterioration by exposure to CS, EC (0% of nicotine) and EC (1.2% of nicotine), as well as the effect of TW. We devised an in vitro model of cell growth at the air-liquid interface for longer exposures. Human bronchial epithelial cells (HBECs) in an air-liquid interface were exposed to a reference CS, and a commercial EC with either 0 or 1.2% nicotine, compared to a clean air control using a normal puffing regime (CORESTA). The HBECs samples were acquired from a commercial entity as primary lung cells isolated from two donors (Table 1, donor 1 and 2), and we uses 3–5 HBECs samples for each of the CS, EC or air only (control group) exposures (Table 1). Differentiated HBECs were exposed to 10 puffs of EC aerosols, CS or air control each day for 10 days. 18 to 24 h recovery periods followed each exposure. Epithelial monolayer function was monitored by FITC-Dextran, TEER, CBF (% of pixels), and cell velocity 16–18 h after the last exposure. To measure the effect on transcript levels, RT-qPCR was performed. An additional set of experiments evaluated the effects of exposure to TW in the epithelial cell barrier function using pseudostratified primary epithelial tissue from donor 3 (MucilAir, Table 1) obtained from a commercial outlet. TW smoke was generated by combustion of commercial tobacco blend (shisha) using the modified Beirut regimen [15]. Three samples from donor 3 in an air-liquid interface were exposed for 1 h every other day until three exposures were performed. To each TW exposure preceded an exposure to the control group (exposed to air only) with a matching number of samples. Every other day refers to a recovery period of 48 h that followed each exposure. Monolayer barrier integrity was evaluated using TEER, CBF (% of pixels), 12–16 h after the last exposure and RT-qPCR was performed following the exposure. The average of 3 to 5 insert samples per experiment allowed us to observe a significant difference among experimental groups using non-parametric statistical analysis. The number of samples analyzed per group is similar to those reported in previous studies [16, 17].

**Table 1:** Number of samples used per donor in each experimental group

Group	CS and EC		TW
Experiment	Donor 1	Donor 2	Donor 3
FITC-Dextran	2	1	N.d.
TEER	3	2	3
CBF and %Pixels	3	2	3
Cell velocity	3	2	N.d.
RT-qPCR	2	1	3
Age	50	40	59
Sex	Male	Female	Female
Health status	Healthy	Healthy	Healthy

We did not collect cells directly; demographic data are provided by Epithelix and MatTek (details in the Methods section), and presented for informative purposes. The number of samples and origin (donor number) in the control groups is matched to information reported in Table 1. N.d, not determined

### Cells

The source of HBECs is MatTek (USA) for EC and CS experiments, and differentiated MucilAir pseudostratified primary epithelial tissue from Epithelix (Switzerland) for TW experiments. The demographic information of the donors, the number of samples for each experiment and each donor are shown in Table 1. Amplification of the cryopreserved HBECs was performed on a collagen-coated flask with PneumaCult-Ex Plus medium at 37 °C, and 5% CO_2_. Passage of cells occurred at 80 to 90% confluency. The density of HBECs was 150,000 cells/well, and 400,000 cells/well for 6.5 mm and 12 mm transwells, respectively, on collagen-coated 0.4 μm pore polyethylene terephthalate, clear membrane transwells with apical and basolateral PneumaCult-Ex Plus medium. HBECs were differentiated for 4 to 6 weeks into pseudostratified epithelia at the air-liquid interface after reaching 100% confluency with PneumaCult-ALI at 37 °C and 5% CO_2_. MucilAir cells were incubated in identical conditions using MucilAir media.

### Smoke and vapor generation and exposure

EC was generated from a commercial V2 vaping pen Pro Series 3-in-1 vaporizer (power 5.19 W, resistance 3.3 Ω) [18] containing a classic tobacco flavor e-liquid cartridge with 0% or 1.2% nicotine (V2), whereas whole CS from reference-grade 3R4F cigarette (Kentucky). The EC and CS exposures were performed according to the Cooperation Center for Scientific Research Relative to Tobacco (CORESTA) Recommended Method No. 81 (CRM N° 81), which consist of a square-wave puff profile of 55 mL puff volume, 3 s duration, and 30 s puff interval [16]. A Masterflex peristaltic pump puffed whole smoke into the Vitrocell (Germany) exposure chamber, whereas the control inserts were exposed to humidified air (control air) in the exposure system using the same profile. In a different set of experiments, we used the modified Beirut profile for TW smoke generation [15]. Briefly, a laboratory-grade waterpipe (Batelle) burned 12 to 13 g of shisha (Exotic Pirate’s Cave, Starbuzz) with a lit 40 mm charcoal placed on top of an aluminum foil (10 min pre-heated) covering the TW head, and smoke generated using a puffing regimen as follow: 530 mL puff, 3 s duration, puff interval 17 s. The charcoal was replaced after 30 min. We omitted the TW head for the control air exposures. The exposure system received whole TW smoke or control air (set to 6 L/min with an on-line manifold), and constant humidified filtered air (1 L/min), while a vacuum input smoke constantly into the exposure chamber at 5 mL/min.

### Permeability measurement

Permeability was assessed by fluorescein isothiocyanate–dextran flux assay (FITC-Dextran flux assay), and trans-electrical epithelial resistance (TEER), as described previously [11] 16 to 18 h post-exposure. For TW exposures, TEER was determined after the second exposure only due to technical issues with TEER measurement of control samples after the third exposure. We did not measure FITC-Dextran flux assay in cells exposed to TW.

### Ciliary beat frequency (CBF)

The plates containing the pseudostratified epithelia were incubated at 37 °C with 5% CO_2_ in the 3iMarianis Spinning-Disk Confocal. High-speed time-lapse videos were taken at 32X air at 100 Hz with a total of 250 frames using a scientific CMOS camera. Five areas imaged per insert. A Matlab (R2018b) script (validated against SAVA) [19] was used to determine average CBF per video, to generate a heat map indicating CBF, and to determine percent pixels moving.

### Cell velocity

Utilizing a microscopy methodology recently developed in our laboratory, we captured time-lapse videos of the epithelial cell monolayer for 2 h following exposure and computed the average velocity for the area. Briefly, on cells exposed to EC or CS, time-lapse videos are taken at phase contrast using the 32X dry objective with one frame taken every 5 min for 2 h. We did not measure cell velocity in cells exposed to TW. Cell migration was quantified by performing Particle Image Velocimetry (PIVlab) on Matlab using multi-pass cross-correlation analysis with decreasing interrogation window size on image pairs to obtain the spatial velocity field as described previously [20].

### *CDH1* expression

Total RNA was isolated from cultured primary bronchial epithelial cells and purified using a column kit supplemented with the Proteinase K and RNase-Free Dnase Set, and cDNA of 1000 ng·μL^− 1^ was obtained using a cDNA Reverse Transcription Kit (Qiagen). In the case of MucilAir cultures exposed to TW, total RNA was isolated with Tryzol and Zymo Clean and Concentrator Kit. The absence of DNA contamination was verified by excluding the reverse transcriptase from subsequent PCR reactions. cDNA was subjected to PCR using the SYBRGreen PCR Master Mix to amplify *CDH1* (E-cadherin), and the housekeeping gene *GADPH* (Glyceraldehyde-3-phosphate dehydrogenase) mRNAs using primers shown in Table 2. *GADPH* is a housekeeping gene in epithelial tissues reliable for *CDH1* quantification [21–24]. The program in the C1000-PCR thermo-cycler was 94 °C, 15 min, followed by 45 cycles (94 °C, 35 s; 60 °C, 1 min; 72 °C, 1 min 15 s) ending in 72 °C, 2 min. Based on the comparative C*t* method, transcript expression levels were calculated. The amplified products were visualized by transilluminator in 3% agarose gel electrophoresis, stained with ethidium bromide.

**Table 2:** DNA primers (5′-3′)

Gene	Forward	Reverse	Product (bp)
*CDH1*	CCCACCACGTACAAGGGTC	CTGGGGTATTGGGGGCATC	94
*GAPDH*	AACGGGAAGCTCACTGGCATG	TCCACCACCTGTTGCTGTAG	304

### Statistical analysis

Prism v7.0 (GraphPad) was used for analysis. As shown in figures, the *p*-values were considered statistically significant (*p* < 0.05) according to a non-parametric hypothesis test. We applied a Kruskall-Wallis rank-sum test followed by Dunn’s multiple comparison test in CS and EC experiments. To compare two data sets in TW, we used a Kolmogorov-Smirnov test.

## Results

### Increased permeability in epithelial cell monolayer exposed to electronic cigarette with nicotine and cigarette smoke

**Fig. 1:**
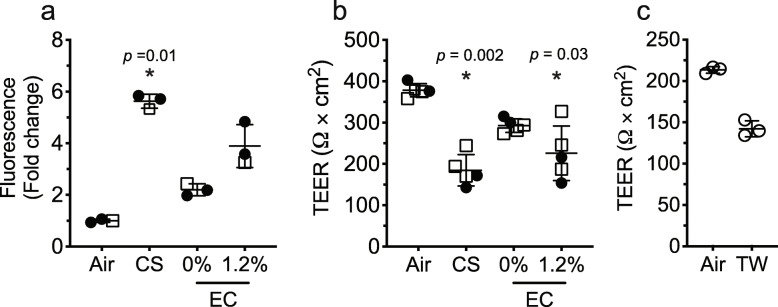
Measurements of permeability in HBECs at air-liquid interface exposed to EC, CS, TW and control air (Air). FITC-Dextran flux assay for EC and CS exposures (**a**), measurement of TEER after exposures to EC or CS (**b**), and after exposures to TW (**c**). Vertical bars are mean (± SD). Significant changes in relation to control air (*). Samples key: donor 1, filled circles (•); donor 2, empty squares (**□**); donor 3, empty circles (ο)

Fig. 1a shows that exposure of HBECs cells to CS significantly increased fluorescence levels, reflecting the loss of barrier function as monolayer permeability increases in the basal media by six-fold compared to the air-exposed monolayer of epithelial cells. Importantly, the monolayer of epithelial cells exposed to 0% nicotine EC aerosol showed a modest increase in permeability, that doubled in cells exposed to EC aerosol with 1.2% nicotine, albeit not significant and ~ 33% less permeable than monolayers exposed to CS.

Figure 1b indicates a trend towards a decrease in TEER of a cell monolayer exposed to EC aerosol with 0% nicotine, compared to control (air-exposed). The presence of nicotine in the aerosol caused a disruption that significantly decreases TEER by ~ 40%, similar to CS. In other set of experiments (Fig. 1c), we showed that in MucilAir cells exposed to TW, TEER dropped by ~ 25% (nonsignificant) compared to baseline after the second exposure. Different basal permeabilities in MucilAir and HBECs hindered direct comparison of toxicological effects; however, EC (0%) and TW tend to decrease TEER. Based on the above results, we demonstrated compromised integrity of the epithelial monolayer barrier due to CS and EC (1.2%) but not EC (0%), suggesting that nicotine plays a major role in this barrier disruption. While the effect is not as dramatic as that with conventional cigarettes, this could be a function of exposure time.

### Exposure to electronic cigarette aerosol with nicotine, and cigarette smoke modulated ciliary beat frequency in an epithelial cell monolayer

**Fig. 2:**
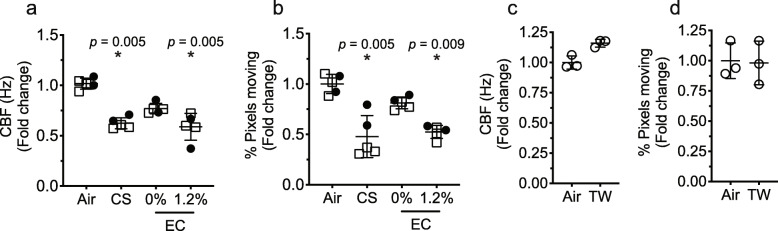
Assessment of CBF in HBECs at air-liquid interface exposed to EC, CS, TW and control air (Air). Shown in **a**-**c**, is CBF fold change relative to control air. 0 and 1.2%, indicates the concentration of nicotine in e-liquid. Panels **b** - **d**, show quantification of the percent of pixels moving. Vertical bars are mean (± SD). Significant changes in relation to control air (*). Donors: donor 1, filled circles (•); donor 2, empty squares (**□**); donor 3, empty circles (ο)

We sought to determine if EC and TW aerosols alter ciliary function. Exposure of cells to EC aerosol resulted in a trend towards a decrease (nonsignificant) in CBF, (Fig. 2a and Additional file 1). While this decrease in CBS was seen without the addition of nicotine, it became significant in the presence of 1.2% nicotine similar to CS exposure. Interestingly, MucilAir cells exposed to TW tend to increase CBF, although nonsignificant. (Fig. 2c).

In addition to beat frequency, the number of beating cilia can also affect mucocilliary clearance; therefore, we quantified this by measuring the number of pixels that were moving in each visual field. We found that EC aerosol exposure tends to decrease pixel counts (nonsignificant), suggestive of fewer beating cilia in the cell monolayer compared to control air, that decreases significantly with 1.2% nicotine in the e-liquid similar to the level observed in cells exposed to CS (Fig. 2b). Furthermore, the unchanged pixel counts in cell monolayer exposed to TW were consistent with the aforesaid tendency to increased CBF (Fig. 2d). The results indicate that only aerosolized EC containing nicotine may decrease epithelial mucociliary clearance, but not those without nicotine.

### Increased cell velocity in epithelial cell monolayer exposed to cigarette smoke or electronic cigarette aerosol with nicotine

**Fig. 3:**
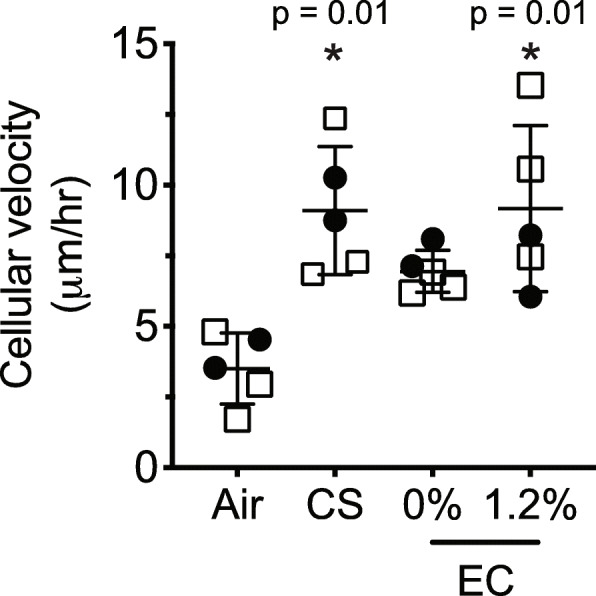
Cell velocity analysis using time-lapse video of epithelial cell monolayer exposed to CS or EC aerosol. We did not measure cell motility in cells exposed to TW. Values show mean ± SD. *, significant changes in relation to control air. Donors: donor 1, filled circles (•); donor 2, empty squares (**□**); donor 3, empty circles (ο)

Using micro-video technology, we capture the movement of cilia and its velocity. Figure 3 shows a significant increase in cell velocity after CS exposure. Furthermore, the EC exposure tends to increase cell velocity that became significant with 1.2% nicotine added, to a similar extend as the CS exposure. (Fig. 3 and Additional file 2).

### Exposure to cigarette smoke dismantled adherence junctions

**Fig. 4:**
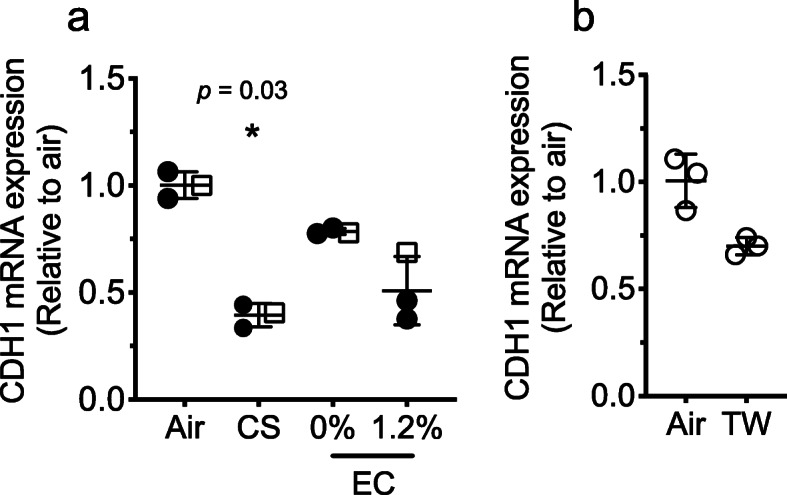
Levels of E-cadherin (*CDH1*) mRNA transcript quantified by RT-qPCR from HBECs exposed to EC, CS, and control air (Air) (**a**) or TW and control air (Air) (**b**). Nicotine concentration in e-liquid is 0% or 1.2%. Vertical bars are mean (±SD). Significant changes in relation to control air (*). Donors: donor 1, filled circles (•); donor 2, empty squares (**□**); donor 3, empty circles (ο)

The exposure of epithelial cells to EC with and without nicotine or TW resulted in a modest decrease (nonsignificant) of *CDH1* transcript (which encodes E-cadherin) compared to the significant decrease (39%) observed in cells exposed to CS (Fig. 4a, b). The decrease in *CDH1* directly correlates with barrier disruption as described above occurring with CS exposure, followed by EC with nicotine. In all experiments, EC (0%) and TW have shown the tendency of disruption, but the observations were not significant, plausible due to the small sample size.

## Discussion

There is increasing recognition that CS can disrupt the epithelial barrier due to the toxicity of the mixture of chemicals acting through several molecular mechanisms that include MAPK (mitogen-activated protein kinase), TGFβ (transforming growth factor beta-1), and reactive oxygen species [25–29]. EC aerosol produced from e-liquid (propylene-glycol and glycerol) and smoke from TW contain many of the same toxicants found in CS; therefore, it is not surprising that similar pathways may be activated by exposure to EC and TW [10, 30–33]. For the determination of toxic effects, we used primary differentiated human lung epithelial cells as they replicate the human lung physiology well enough for assessments of health risks [34]. Two cell models were used for CS-EC and TW. Therefore, we minimized comparisons across the two sets of experiments to account for functional differences in cell barrier permeability observed in this study and as reported by other researchers [17]. The changes in fluorescence levels that resulted from CS exposure (FITC-Dextran assay decreased 5.6 times, and TEER decreased to 50% compared to control air) are in line with the previously observed loss of permeability after CS exposure [8, 11]. In the exposure to EC with or without nicotine, we observed a trend to decreased permeability with FITC-Dextran assay and when using TEER to measure barrier properties, the toxic effect of EC (1.2%) became significant, decreasing permeability by 60% compared to air control. TEER measures the movement of ions across an electric gradient applied parallel to the monolayer, with a decreased resistance reflecting a loss of monolayer integrity [35]. A significant change of TEER in cells exposed to TW may be masked by a low number of samples. Nevertheless, our previous studies indicate that the permeability of epithelial monolayers increase only after repeated exposure to cigarettes, suggesting that disruption of permeability, as observed in this study, is in proportion to the length of the exposure [11, 16, 36]. Indeed, an absence of epithelial barrier disruption in in vitro models of very short exposures to EC has been reported [37]. In addition, flavors in the e-liquid may positively modulate permeability as well, which was previously shown in cell lines exposed to EC [38]. In our exposure with EC tobacco flavor without nicotine we do see a tendency to a reduction in barrier integrity based on TEER measurements that is significant in the presence of nicotine. Furthermore, we demonstrated that the presence of nicotine in EC (1.2%) deteriorates mucociliary clearance by decreasing CBF by 59% with respect to the control air. Mucociliary clearance mechanism is one of the critical protective functions of the epithelial monolayer that is performed by the rhythmic and directional beating of cilia on a significant subpopulation of the monolayer [39]. Previous studies have shown shortening or loss of cilia, as well as decreases in CBF, are a direct result of the volatile organic compounds [40–44]. Whereas CS has a high concentration of volatile organic compounds, EC aerosols have concentrations that vary with the abundance of flavoring components and the glycerol:propylene-glycol ratio in the e-liquid [45–48]. Here, we show a significantly decreased CBF and pixel movement upon exposure to CS or EC (1.2%) by 62 and 47%, respectively. We and other researchers have shown that CS exposure leads to decreased CBF and number of cilia in epithelial cell monolayers that is in line with previous studies in which deteriorating effects of CS on ciliated cells were reported [11, 13, 49]; however, the results obtained from our chronic EC (1.2%) exposure are novel. The plausible additive effect of nicotine in decreasing CBF, suggested in our experiment, is in striking contrast with recent report that showed nicotine exposure of cultured epithelial cells has no effect on CBF [50]. Therefore, based on our results, a synergistic effect of nicotine and additional components of EC such as flavors, as well as other components of EC, need to be tested specifically in settings of the aerosolized EC exposure. For example, nicotine showed an additive effect in decreased mucociliary clearance mechanism and other lung epithelial lesions in cells and in vivo experiments of EC exposures [51, 52].

We have identified specific trends in effect with TW, albeit with weak statistical power due to a small sample number (3 replicas) in the TW experiments. While this does hamper any comprehensive conclusions, we do believe that this in vitro model is ideal for sub-chronic exposures and can be adapted for chronic exposures of TW and other tobacco products in need of urgent toxicological assessment (e.g. EC) [53, 54]. We note that whereas a (nonsignificant) increase in CBF may be a distinct effect of TW in our study, is not unprecedented. We have recognized several instances of similar responses to CS in vitro [42, 55], ex-vivo [56, 57], *and* in vivo [58]. Moreover, we have previously shown that continuous monitoring of CBF in lung epithelial cells exposed to CS revealed temporal fluctuations, with the appearance of CBF curve inflections in cells, minutes and hours into the recovery time [20]. Thus, while it could be attributed to distinct components present in the TW smoke [59], it is also entirely plausible that chronic exposures to TW would cause decreases in CBF, similar to that seen with EC and CS. We are currently planning to test this in our laboratory.

We have previously demonstrated a 5 to 10 times increase in cellular velocity of epithelial cells exposed to CS compared to control [20], and here we showed evidence of 2.5 and 2.6 times increase after exposure to CS and EC (1.2%), respectively. The underlying molecular mechanisms that control cell velocity after CS or EC (1.2%) exposures are beyond the scope of this report, and it will be examined in the future, but this observation correlates with the loss of cell-cell adhesion proteins and altered actin-myosin contractility [11]. Nevertheless, accumulating evidence suggests that a decreased expression of the *CDH1* gene, the main component of adhesin junctions in lung epithelial cells, is a marker of the loss of barrier function in response to CS [60, 61]. Here, we show that EC (1.2%) tended to decrease *CDH1* expression, while CS decreased by 39% compare to the control air. Therefore, future work is needed to explore if exposure to EC (1.2%) alike that of CS, impairs the stability of adherence junctions that are necessary to keep adequate cell-cell interactions in the lung epithelium.

## Conclusions

We devised a sub-chronic exposure system to measure the toxic effects of EC and other tobacco products by monitoring cell barrier disruption. Our results, albeit with limited number of samples, support that sub-chronic exposure to EC with 1.2% nicotine may disrupt the airway epithelia, similar to CS. More research with larger number of donor samples is being planned for the future. While, in our experimental conditions, EC 0% and TW caused less epithelial barrier disruption, future experiments need to address the toxicological significance of prolonged exposure and nicotine concentration as well as other components of these tobacco products as determinants of harmful effects. Specifically, for EC and TW, the addition of a variety of flavors needs to be addressed, as they are not proven safe when inhaled. Our system is adequate for future studies of chronic exposures that are required to understand the health effects of long-term users of EC and TW.

## Supplementary information

**Additional file 1.** Exposure of epithelial cell monolayer to EC or CS shows dysregulation of CBF. The heat map is an overlap of a single field of view of HBECs at ALI. Hz (Hertz). Panels: control air (a), EC aerosol with 0% nicotine (b), EC aerosol with 1.2% of nicotine (c), CS (d).

**Additional file 2.** Showed is the heat map analysis of a single image taken during recording time-lapse video of HBECs exposed to (left to right) control air, CS, EC (0%) nicotine and EC (1.2%) nicotine.

## Data Availability

This published article and its supplementary information files include all data generated or analyzed during this study. Raw data is available upon submission of request to vsidhay1@jhmi.edu, hreyesca@gmail.com or sbiswaljh@gmail.com.

## Abbreviations

CBF: Cilia beat frequency
COPD: Chronic obstructive pulmonary disease
CS: Cigarette smoke
EC: Electronic cigarettes
HBECs: Primary human bronchial epithelial cells
MAPK: Mitogen-activated protein kinase
TEER: Trans-electrical epithelial resistance
TGFβ: Transforming growth factor beta 1
TW: Tobacco waterpipe
