# Supplementary material for: Effect of sub-chronic exposure to cigarette smoke, electronic cigarette and waterpipe on human lung epithelial barrier function
Source: BMC Pulm Med. 2020 Aug 12;20:216. doi: 10.1186/s12890-020-01255-y (PMC7425557; doi:10.1186/s12890-020-01255-y)
Supplement: Supplementary file 1 — Additional file 1. Exposure of epithelial cell monolayer to EC or CS shows dysregulation of CBF. The heat map is an overlap of a single field of view of HBECs at ALI. Hz (Hertz). Panels: control air (a), EC aerosol with 0% nicotine (b), EC aerosol with 1.2% of nicotine (c), CS (d). [file 12890_2020_1255_MOESM1_ESM.docx]

**Additional file**


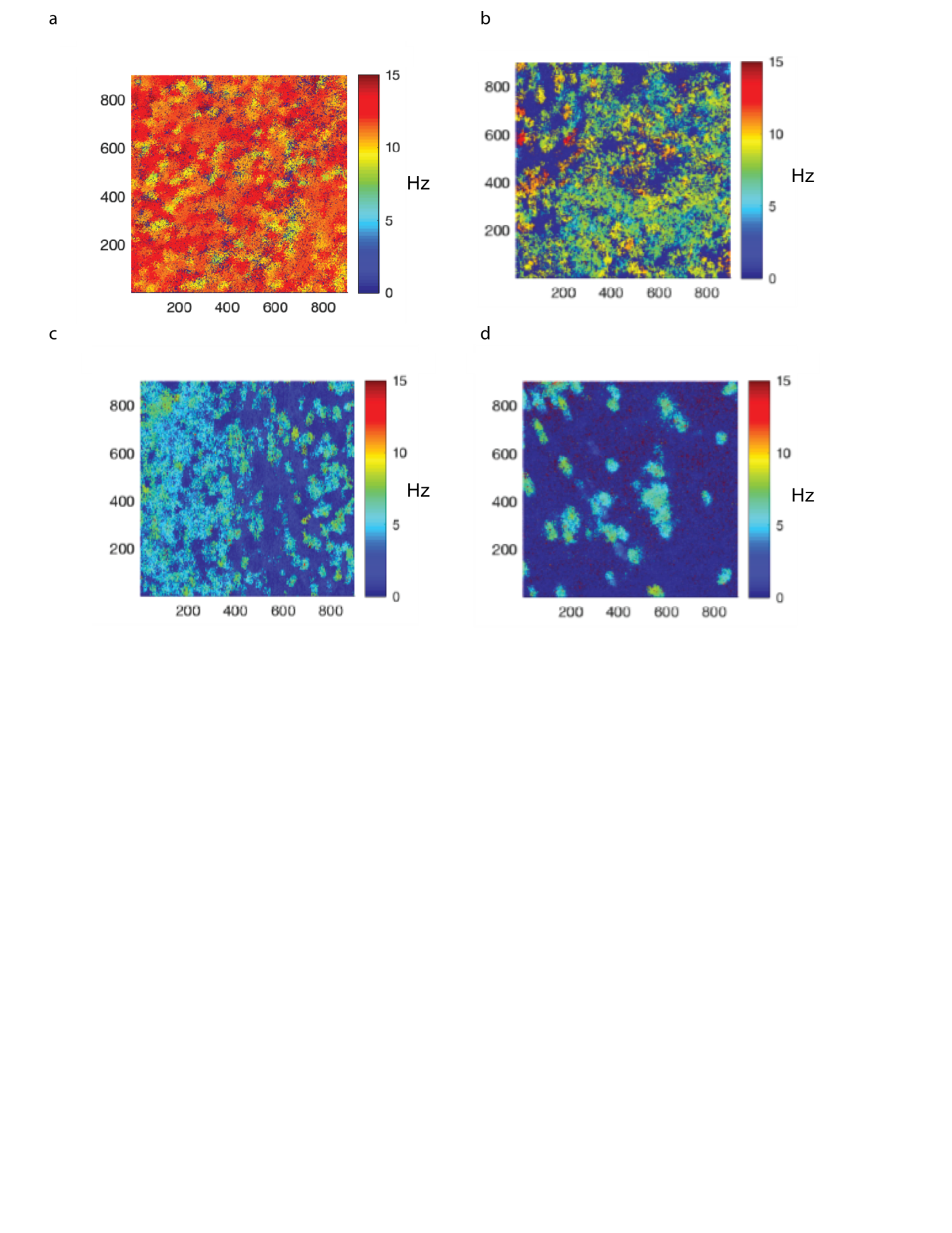


Additional file 1 Exposure of epithelial cell monolayer to EC or CS shows dysregulation of CBF. The heat map is an overlap of a single field of view of HBECs at ALI. Hz (Hertz). Panels: control air (a), EC aerosol with 0% nicotine (b), EC aerosol with 1.2% of nicotine (c), CS (d).
