# Supplementary material for: Effect of sub-chronic exposure to cigarette smoke, electronic cigarette and waterpipe on human lung epithelial barrier function
Source: BMC Pulm Med. 2020 Aug 12;20:216. doi: 10.1186/s12890-020-01255-y (PMC7425557; doi:10.1186/s12890-020-01255-y)
Supplement: Supplementary file 2 — Additional file 2. Showed is the heat map analysis of a single image taken during recording time-lapse video of HBECs exposed to (left to right) control air, CS, EC (0%) nicotine and EC (1.2%) nicotine. [file 12890_2020_1255_MOESM2_ESM.docx]

**Additional file**


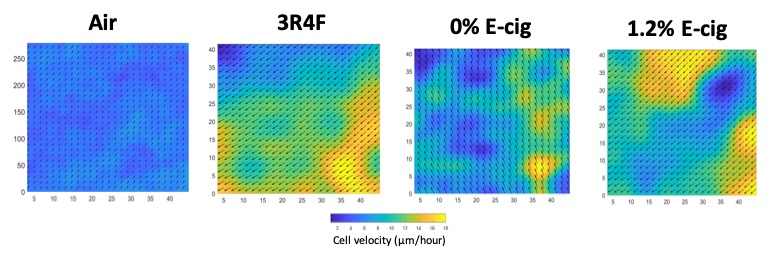
Additional file 2 Showed is the heat map analysis of a single image taken during recording time-lapse video of HBECs exposed to (left to right) control air, CS, EC (0%) nicotine and EC (1.2%) nicotine.
